# Implementing Telemedicine Intervention in Neonatal Intensive Care Units: Augmented Teleconsultation and Real-Time Monitoring Experience

**DOI:** 10.1089/tmr.2024.0088

**Published:** 2025-02-13

**Authors:** Dalia M. Mominkhan, Faisal Aldahmashi, Ali H. Almudeer, Abdulaziz S. Alhmod, Muaddi F. Alharbi, Lamya M. Alzubaidi, Nada K. Alwehaibi, Khalid N. Alobeiwi, Manea M. Balharith, Ahmed A. Alahmari, Fahad A. Alamri, Ghadah Alsaleh, Yaser Almuzaini, Mohammed K. Alabdulaali

**Affiliations:** ^1^ National Health Command Center, Ministry of Health, Riyadh, Saudi Arabia.; ^2^ Assistant Deputyship of Hospital Affairs, Ministry of Health, Riyadh, Saudi Arabia.; ^3^ Neonatology Department, King Fahd Central Hospital, Jazan, Saudi Arabia.; ^4^ SEHA Virtual Hospital, Ministry of Health, Riyadh, Saudi Arabia.; ^5^ The Studies and Consulting Office at the Assistant Minister of Health, Ministry of Health, Riyadh, Saudi Arabia.; ^6^ National Health Command Center, Ministry of Health, Riyadh, Saudi Arabia.; ^7^ National Health Command Center, Ministry of Health, Riyadh, Saudi Arabia.; ^8^ Hospital Administration Department, Ministry of Health, Riyadh, Saudi Arabia.; ^9^ National Health Command Center, Ministry of Health, Riyadh, Saudi Arabia.; ^10^ Global Centre for Mass Gatherings Medicine, Ministry of Health, Riyadh, Saudi Arabia.; ^11^ Assistant Minister of Health, Ministry of Health, Riyadh, Saudi Arabia.

**Keywords:** bed occupancy, intensive care unit, mortality rate, neonates, teleconsultation, telemedicine

## Abstract

**Background:**

Increasing intensivist shortages and demand, coupled with the escalating bed occupancy rate due to increased demand for neonatal intensive care units (NICUs), have created enthusiasm for tele-critical care (TCC) in the form of teleconsultations. Consequently, this study aimed to describe the role of TCC intervention in enhancing NICU capacity to manage discharge, bed occupancy, and neonatal mortality rates.

**Methods:**

This was an uncontrolled, retrospective, interventional descriptive study conducted over 22 months from January 2021 to October 2022 in a public hospital in Najran, Saudi Arabia. We employed the scheduled care model of TCC, in which an intensivist provides daily rounds, overnight calls, and critical care consultations upon request. Real-time outcomes, including mortality, discharge, and bed occupancy rates, were monitored in real-time by the National Healthcare Command Center.

**Results:**

Implementing the TCC program was associated with an overall reduction of 10.7% in the neonatal mortality rate from 10.3 to 9.2 deaths per 1000 live births. The discharge rate increased from 0% in the early months of the TCC application to 34.12% after 4 months of application despite the increased bed occupancy rate. The study revealed no statistically significant difference in mortality rates between the means of pre- and post-TCC (M = 9.74, SD = 4.32), (M = 10.28, SD = 7.99) respectively, *p* = 0.856 with a 95% confidence interval of −5.58 to 6.66.

**Conclusions:**

TCC in virtual scheduled consultations with a real-time dashboard was proven successful in controlling neonatal mortality and discharge rates. Further studies are required with extended follow-up periods and involving parameters such as the acceptance of physicians, long-term effects beyond the NICU, and the impact of TCC on logistics and resources.

## Introduction 

Policymakers aim to improve the quality of care and efficiency of health systems. Aligning both objectives may be challenging, and a trade-off may arise between efficiency and quality of care.^1^ Within the hospital sector, the bed occupancy rate, which is “the ratio of the number of occupied beds to available beds,” is a major concern that might affect efficiency and quality.^2^ Hospital occupancy rates are rising because of rising demand for care and declining bed availability. Premature discharge, overcrowding of facilities, increased staff workload, and diminished quality of care are potential outcomes of high bed occupancy rates, which may indicate a stressed health care system.^3^ Moreover, several studies have proposed a significant association between high occupancy rates and inpatient mortality.^4,5^ Conversely, low bed occupancy may indicate underutilization and provide an opportunity to enhance productivity.^3^

Preterm infants born at less than 33 weeks gestational age have a higher risk of neonatal complications and require more time in neonatal intensive care units (NICUs) than full-term infants.^6^ Recent studies have shown significant variations in length of stay (LOS) in NICUs between countries.^7–9^ Variations in NICU - LOS may be caused by several factors, including access to step-down units, parental education quality, and post-discharge follow-up structure.^10^ A well-functioning, regionalized perinatal care system requires not only tertiary NICU beds but also beds in step-down units (level 2); thus, convalescent infants may receive the appropriate degree of care closer to home.^11^ However, it is important to maintain flexibility to meet the daily fluctuations in neonatal care demands. Bed occupancy rates between 75% and 80% are considered ideal and safe estimates for NICU components.^12^ Operating a tertiary NICU above that point results in serious issues such as nosocomial infections and a worse survival rate.^13,14^

In addition, it is unlikely that daily variations in bed availability at secondary and tertiary levels would increase. To control such fluctuations, well-trained and prepared teams of health care providers, including general practitioners, specialists, consultants, and nurses, are required. Mortality rates, hospital stays, and patient satisfaction improve when experienced physicians assist adult and pediatric intensive care units (ICUs) .^15–17^

To address these challenges, health care systems should utilize appropriate resources in their neonatal care services to accommodate daily fluctuations in demand while maintaining safe occupancy levels. This approach requires careful planning, resource allocation, and ongoing monitoring to ensure that preterm infants receive the best care throughout and beyond their NICU stay. Adherence to best practices in critical care has been linked to better clinical outcomes, lower death rates, and shorter hospital stays, as shown by Lilly et al.^18^ Recently, telemedicine has shown promising results in preparing teams to adhere to best practices through what is called virtual consultations.

Telemedicine is providing medical services remotely with the assistance of telecommunications. These can include video calls, phone calls, and other types of remote communication.^19^ This emerging digital solution allows for remote diagnosis and treatment of patients, and it has the benefit of receiving care remotely from a doctor who is often more specialized than the patient’s general practitioner, making telemedicine an ideal fit for chronic illnesses requiring ongoing care monitoring.^20^ Telemedicine is also ideal for bedridden patients or patients with limited mobility, patients living in rural areas, or patients traveling abroad, enabling easy access to medical care.^21^ Telemedicine and teleconsultations can also be more affordable options for patients with chronic illnesses, such as diabetes, asthma, or cardiac disease.^22^

Many investigators have explored the application of teleconsultations in NICUs in different countries. In Brazil, Melo et al. showed that using the telemonitoring system in NICUs was associated with improved qualification of the involved professionals, care support, adhesion to best practices, reduction of displacements and transferences, and regulation of beds.^23^ Similarly, Makkar et al. showed that teleconsultation application in a regional level III NICU in the USA was associated with a 5.4-day reduction in the NICU LOS of low-birth-weight infants.^24^

In Saudi Arabia, the birth rate declined from 17.562 to 16.166 births per 1000 people between 2019 and 2022. Likewise, the neonatal mortality rate decreased from 6.103 to 5.497 deaths per 1000 live births between 2019 and 2022, which is projected to be reduced to 2.936 per 1000 live births by 2050.^25^ However, the neonatal mortality rate in Saudi Arabia is still higher than that in some neighboring countries, such as the United Arab Emirates and Bahrain (4.936 and 5.290 per 1000 live births, respectively). Therefore, continued efforts to reduce the neonatal mortality rate are required.

The health care system provides services without charge for the majority of Saudi citizens through public health facilities, while employees in the private sector receive medical care through mandatory health insurance via private health facilities. The Saudi Ministry of Health exercises regulatory oversight over all health care services within its jurisdiction. The use of telemedicine has substantially increased in Saudi Arabia, particularly following the COVID-19 pandemic, which has involved outpatient clinics, consultation services, call centers, remote monitoring, and mobile applications. In 2019, the Saudi Ministry of Health established the Saudi Telemedicine Unit of Excellence and allocated annual operational funds to prompt the broad implementation of virtual consultation services across the Kingdom and to provide regulations for their use. Besides, training programs are frequently conducted for a better understanding of telemedicine services among providers.^26^ In this study, we aimed to describe the role of TCC intervention in enhancing NICU capacity in a secondary center in Saudi Arabia in reducing discharge, bed occupancy, and neonatal mortality rates.

## Methods

This uncontrolled, retrospective, interventional descriptive study was conducted over 22 months after the study was performed in a public hospital in Najran, Saudi Arabia. As a result of the increasing bed occupancy and neonatal mortality rates in January 2022 in the hospital in Najran, Saudi Arabia, a ministerial committee of 11 experts was formed to investigate the causes of these increasing trends and check the readiness and preparedness of the hospital’s managerial team and health care providers. Moreover, the committee reviewed the policies, procedures, and protocols and compared them with international and national guidelines. This study was approved by the ethics committee of the Institutional Review Board of the King Fahad Medical City, Riyadh, Saudi Arabia, IRB, log no. 24-240E on 28 May 2024.

Following an extensive situation analysis and review of epidemiological factors, causes, and sources of infections, the committee identified several challenges impeding better control of bed occupancy and neonatal mortality rates, including (1) inadequacies in following up improvement projects and recommendations; (2) shortcomings in the operational role of family management and transfer; (3) nonimplementation of procedures and policies, and the staff’s lack of knowledge thereof; (4) insufficient effective communication between internal departments; (5) public resistance to medical referrals to public and private sector hospitals; (6) increased resignations in the neonatal nursing department; (7) enhancement in the obstetrics and gynecology emergency department, which has resulted in overcrowding; and (8) a shortage of consultants and pediatric specialists in the hospital. The committee proposed several recommendations based on these challenges, including improving entrance and exit policies, internal communication, and primary education courses. Moreover, they emphasized the necessity of implementing all policies and procedures, including the Do Not Rescue policy and new referral protocols. In addition, they suggested the implementation of teleconsultations to address shortages of health care personnel.

**FIG. 1.**
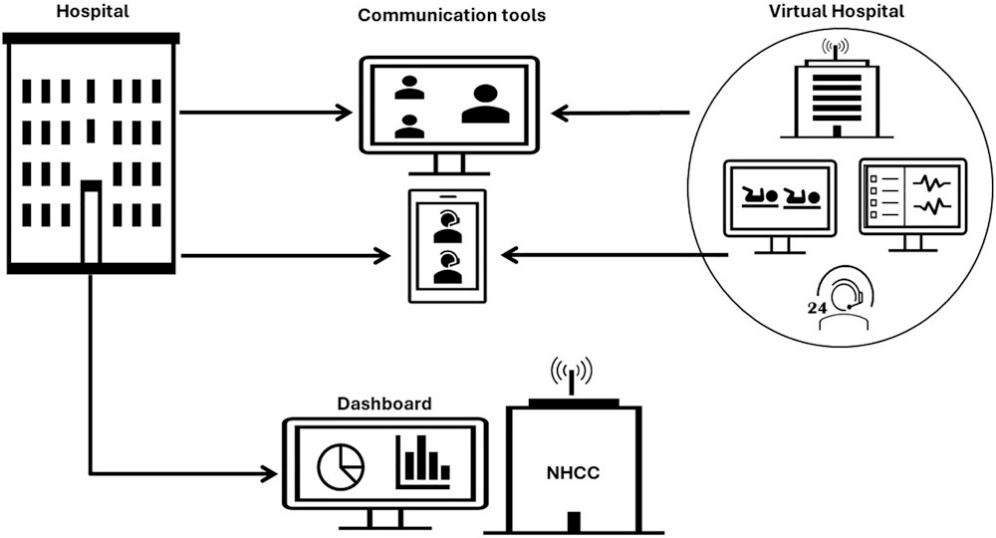
The tele-critical care (TCC) model in the Saudi Ministry of Health.

Based on these recommendations, we have employed the TCC system or Seha “Health” Virtual Hospital, which consists of tele adult ICU, pediatric ICU, and NICU. TCC involves using telemedicine in ICUs and technology to assist in providing care for critically ill patients through off-site clinical resources. This system enables clinicians to quickly access a second opinion on complex cases and develop the most appropriate care plan for patients. In addition, it was designed to ensure that there was appropriate training of clinical staff so that patients consented, were informed, and had access to their clinicians of choice with appropriate safeguarding measures and information security. Clinicians can interact seamlessly with each other between the virtual hospital, the spoke hospital, and the spoke hospital patient, and primary health care physicians can interact with the hospital without the need for avoidable travel or loss of time from work to attend a physical hospital appointment. Hospital ICUs are connected to intensivists and other critical care consultants through networks of audiovisual communication and computer systems. Through these networks, staff interactions can be facilitated via video, phone, or online computers as well as remote real-time monitoring of vital signs and chronic conditions, as illustrated in Figure 1. Video cameras enable telemedicine practitioners to observe the equipment and monitors in the patient’s room. Cameras have an alert system to announce that TCC staff are in visual contact to share observations and care recommendations with bedside caregivers.

There are three models for the TCC system: continuous monitoring, scheduled care, and responsive care. In this study, we applied the scheduled care model in which an intensivist provided daily rounds and critical care consultations upon request and was not responsible for continuously monitoring all units. In this model, a mobile cart with a video-audio platform utilizing Veedoc provided by Al Faisaliah Medical Systems Company, Riyadh, Saudi Arabia, was used to gather information, followed by treatment recommendations given by the consultant. Hospitalists or internists can review the care plans of new admissions with a tele-intensivist by phone or via the audio-video interface from the patient’s room. The medical team responsible for tele-intensive care was divided into two teams, each comprising five consultants specialized in the NICU. The tele-intensivist conducted regular daily morning rounds. After hours, the tele-intensivist can be called by the physician or bedside the registered nurse for help, just as an on-call physician would be called at home. The tele-intensivist can also assist surgeons in managing patients postoperatively. Finally, real-time monitoring indicators were passed through the National Healthcare Command Center (NHCC) of the Ministry of Health to monitor improvements in mortality, discharge, and bed occupancy rates in the unit.

## Results

### Neonatal mortality rate

Before the application of teleconsultations (from January 2021 to February 2022), the total number of births was 8436, and the total number of deaths was 82, with an infant mortality rate of 10.3 deaths per 1000 live births. The peak mortality rate was observed in January 2022 (32.67 deaths per 1000 live births), followed by March and February 2021 (13.49 and 12.73 deaths per 1000 live births, respectively). The neonatal mortality rate in January 2022 is approximately five times higher than that in January 2021. After the application of teleconsultations (starting from February 2022), the total number of births was 5096, and the total number of deaths was 50, with a neonatal mortality rate of 9.2 deaths per 1000 live births. The peak of deaths was observed in March 2022 (19.93 deaths per 1000 live births), followed by July 2022 (12.5 deaths per 1000 live births).

Compared to pre-teleconsultations, the infant mortality rate declined by 10.70%. Between January and February 2022, the neonatal mortality rate decreased by 74.37%. On a monthly level, a reduction in the infant mortality rate was observed in February 2022 (34.20%), May 2022 (34.72%), August 2022 (8.38%), and October 2022 (33.64%) compared with the same month in 2021. On the contrary, we observed that the neonatal mortality rate increased in some months in 2022 compared with the same month in 2021, such as March (32.30%), April (82.5%), June (48.40%), July (40.39%), and September (16.02%). An independent *t*-test was conducted to determine whether there was a significant difference in means between pre- and post-teleintervention on the monthly mortality rate. The results indicated no statistically significant difference between pre- and post-TCC on the mortality rate, *t*(20) = 0.184, *p* = 0.856, with the 95% confidence interval of the difference between means ranging from (−5.58 to 6.66). Although the mortality rate of post-TCC (M = 9.74, SD = 4.32) was lower than pre-TCC (M = 10.28, SD = 7.99), the mean difference was 0.54 (Table 1).

**Table 1.** Independent *t*-Test of the Means of Mortality Rate between Pre-and post-TCC

	*N*	Mean	Std. deviation	Std. error mean
Pre-TCC	13	10.28	7.99	2.21
Post-TCC	9	9.74	4.32	1.44

	Mean difference	Std. error difference	95% CI of the Difference		*t*	df	Significance two-sided *p*
			Lower	Upper			
Pre-TCC and Post-TCC							
Equal variances	0.54	2.93	−5.58	6.66	0.184	20	0.856
Unequal variances	0.54	2.64	−4.99	6.07	0.20	19.15	0.840

TCC, tele-critical care.

### Bed occupancy

In January 2021, the bed occupancy rate was 100%, increasing to 137% in February, 108% in March, 127% in May, and 135% in June. However, in May and June, the discharge rate increased from 0% in the previous months to 34.12% and 16.43%, respectively, despite the increased bed occupancy rate.

## Discussion

The present study demonstrated that the implementation of teleconsultation was associated with a reduction in neonatal mortality and an increase in discharge decisions. However, the impact of teleconsultations on the bed occupancy rate was not changed. Our findings regarding teleconsultation underscore its role in enhancing clinical judgment and decision-making among participants, which may be further reflected in subsequent months due to the cumulative effect of knowledge and experience acquisition. The role of real-time performance monitoring by the NHCC improved outcomes through immediate intervention and direct access to the hospital database. Furthermore, teleconsultation presents opportunities for several improvements in health care sector management, potentially reducing costs associated with additional personnel recruitment and mitigating the risk of human errors based on individual judgment.

Similar results have been reported in several studies conducted across various countries. Kim et al. implemented the TCC in NICUs in Arkansas, USA, and observed a statistically significant reduction in the percentage of very low birth weight deliveries from 13.1% to 7.0% (*p* = 0.01), which corresponded to a decrease in the neonatal mortality rate from 8.5 to 7 deaths per 1000 live births.^27^ Regarding NICU LOS, Rendina et al. showed that telecardiology consultations reduced NICU LOS by over 17%, which was more significant in very low birth weight neonates.^28^ McLeroy et al. observed that the TCC program was associated with increased hospital and ICU patient volume, surgical patient volume, and patient complexity,^29^ which may elucidate our study’s elevated bed occupancy rates.

Regarding the role of TCC in the ICUs, a meta-analysis of 13 studies demonstrated a significant reduction in ICU mortality and LOS following the implementation of TCC (odds ratio [OR]= 0.80; 95% confidence interval [CI], 0.66 to 0.97; *p* = 0.02) and (mean difference [MD]= −1.26 days; 95% CI: −2.21 to −0.30; *p* = 0.01). However, the reduction was not statistically significant regarding in-hospital mortality or LOS (*p* = 0.08 and 0.16, respectively).^30^ A more recent meta-analysis of 19 studies demonstrated that TCC was associated with a significant reduction in ICU LOS (MD = −0.63 days; 95% CI: −0.28 to −0.17; *p* = 0.007), hospital mortality (OR = 0.74; 95% CI: 0.58 to 0.96; *p* = 0.02), and ICU mortality (OR = 0.83; 95% CI: 0.72 to 0.96; *p* = 0.01).^31^ On the contrary, a systematic review conducted in 2016 by Mackintosh et al. evaluated the effects of tele-ICU programs with 24/7 decision support and revealed a reduction in hospital mortality (OR = 0.40; 95% CI: 0.31 to 0.52).^32^ One of these studies included baseline and follow-up data from 38 hospitals and 56 adult ICUs and showed that TCC was associated with lower LOS and mortality rates.^32^ Using CMS data from 2001 to 2010, Kahn et al. compared 132 hospitals with TCC and 389 without TCC.^33^ After adjusting for factors such as hospital size, patient demographics, and location, they found that ICUs that adopted TCC had a lower 90-day mortality rate (OR = 0.96; 95% CI: 0.95 to 0.98; *p* = 0.001). Interestingly, hospitals with the highest reduction in 90-day mortality were more likely to have high volumes and urban locations.^33^

In evaluating the potential improvement of resource allocation in a health system with limited funding through TCC, cost-effectiveness analysis is crucial. Yoo et al. (2016) conducted an assessment of the incremental cost-effectiveness ratio (ICER) of a tele-ICU from the hospital system perspective, utilizing a standard decision model based on available literature.^34^ Quality-adjusted life years (QALYs) accumulated over 5 years post-ICU discharge were utilized to measure cost-effectiveness. In comparison to ICUs without telemedicine, the model predicted that tele-ICUs would contribute an additional 0.011 QALYs at an incremental cost of $516 per patient, resulting in an ICER of $45,320, 95% CI (−$229,016 to $375,870) per additional QALY, which reflects the substantial variability in key outcomes among the published studies.

The efficacy of TCC is contingent upon clinician acceptance. Acceptance studies have yielded conflicting results regarding the potential for conflict between bedside physicians and TCC professionals and the perceived increased strain and burdens associated with continuous monitoring. Subsequent to implementing a tele-ICU and establishing effective communication and utilization patterns among teams, these concerns were frequently mitigated.^35^ Young et al. conducted a systematic review of 23 studies on the adoption of tele-ICU and found that 82.3% to 100% believed that having access to TCC improved the quality of care.^36^ In addition, over 60% of the residents who completed their training in an ICU with telemedicine assistance expressed a desire to continue working in ICUs with such programs after finishing their residency.

The potential of tele-ICUs to diminish the necessity for patient transfers and excessive utilization of tertiary care facilities has been postulated, as they enable ICUs with constrained resources to provide adequate patient care. Multiple investigations suggest that tele-ICUs can augment the capabilities of resource-limited ICUs, potentially reducing transfer requirements.^37,38^ Nevertheless, this was questioned by Pannu et al., who revealed that TCC programs lead to more interhospital transfers from ICUs with fewer resources; this was independent of patient disease severity.^39^ Similarly, no increase in transfers from ICUs with high-intensity coverage has been seen in the Cleveland Clinic experience,^40^ while another study found that monthly transfers to a high level of care decreased from 63 to 57 after implementing a virtual ICU program.^37^

Clinical and economic results have been the subject of investigations in tele-intensive care units. The effects on hospital operations, logistics, and support outside the ICU, such as rapid-response teams, have not been studied. Some personnel outcomes might be necessary, including career extension for clinicians unable to continue bedside work due to injury or illness, backup resources for less experienced bedside clinicians, and intensivist and nurse job satisfaction. These outcomes are crucial because burnout often exhausts the ICU crew and worsens supply shortages.^41^ Ultimately, the impact of tele-ICUs on transfers and resource utilization may depend on various factors, including the specific implementation model, local health care system structure, and capabilities of participating ICUs.^38^

We acknowledge that our study has certain limitations, including the inability to adjust for potential confounding variables, which may limit the generalizability of our data and sociodemographic variables to assess the effects of sex and age. Nevertheless, our study provides a valuable perspective on the implementation in a country that has not been extensively explored in previous research.

## Conclusion

Implementing TCC in virtual consultations with oversight from the NHCC has been proven to be a successful method for controlling infant mortality and discharge rates. Telemedicine facilitates easy access to critical neonatal cases in remote hospitals, provides opportunities for secondary opinions, and enables sharing of management plans. Innovative practices should promote these advantages. This experience can be applied to countries with advanced digital health technologies. As telemedicine transforms management in intensive care units, further investigations are necessary with an extended follow-up period involving parameters such as the acceptance of physicians, long-term effects beyond the ICU, and the impact of TCC on logistics and resources.

## Ethical Approval

This study was approved by the King Fahad Medical City IRB, log no. 24-240E on 28 May 2024. The privacy and confidentiality of patient data were ensured throughout the study, and the primary investigator only accessed it without identifiers.

## Data Availability

The data collected and used in this study are available from the corresponding author upon request.

## Abbreviations Used

ICER =: incremental cost-effectiveness ratio
ICU =: intensive care unit
LOS =: length of stay
NICU =: neonatal intensive care unit
NHCC =: National Healthcare Command Center
QALY=: Quality-adjusted life year
TCC =: tele-critical care
